# Evaluation of In Vitro Activity of Double-Carbapenem Combinations against KPC-2-, OXA-48- and NDM-Producing *Escherichia coli* and *Klebsiella pneumoniae*

**DOI:** 10.3390/antibiotics11111646

**Published:** 2022-11-17

**Authors:** Lisa Allander, Karin Vickberg, Pernilla Lagerbäck, Linus Sandegren, Thomas Tängdén

**Affiliations:** 1 Department of Medical Sciences, Uppsala University, 751 85 Uppsala, Sweden; 2 Department of Medical Biochemistry and Microbiology, Uppsala University, 751 23 Uppsala, Sweden

**Keywords:** carbapenem resistance, Gram-negative bacteria, combination therapy, synergy

## Abstract

Double-carbapenem combinations have shown synergistic potential against carbapenemase-producing *Enterobacterales,* but data remain inconclusive. This study evaluated the activity of double-carbapenem combinations against 51 clinical KPC-2-, OXA-48-, NDM-1, and NDM-5-producing *Escherichia coli* and *Klebsiella pneumoniae* and against constructed *E. coli* strains harboring genes encoding KPC-2, OXA-48, or NDM-1 in an otherwise isogenic background. Two-drug combinations of ertapenem, meropenem, and doripenem were evaluated in 24 h time-lapse microscopy experiments with a subsequent spot assay and in static time-kill experiments. An enhanced effect in time-lapse microscopy experiments at 24 h and synergy in the spot assay was detected with one or more combinations against 4/14 KPC-2-, 17/17 OXA-48-, 2/17 NDM-, and 1/3 NDM-1+OXA-48-producing clinical isolates. Synergy rates were higher against meropenem- and doripenem-susceptible isolates and against OXA-48 producers. NDM production was associated with significantly lower synergy rates in *E. coli*. In time-kill experiments with constructed KPC-2-, OXA-48- and NDM-1-producing *E. coli*, 24 h synergy was not observed; however, synergy at earlier time points was found against the KPC-2- and OXA-48-producing constructs. Our findings indicate that the benefit of double-carbapenem combinations against carbapenemase-producing *E. coli* and *K. pneumoniae* is limited, especially against isolates that are resistant to the constituent antibiotics and produce NDM.

## 1. Introduction

The emergence of carbapenemase-producing *Enterobacterales* presents a major medical challenge. Carbapenem-resistant bacteria typically display a multidrug-resistant phenotype and are associated with prolonged hospitalization and high mortality rates [1,2,3]. Combination therapy is often employed for these infections to enhance the activity of available antibiotics and improve patient outcomes [4].

Carbapenems are often considered last-resort agents against Gram-negative bacteria due to their broad spectrum of activity and inherent stability against most β-lactamases [5]. However, the activity of these antibiotics is hampered by carbapenemases, which hydrolyze virtually all β-lactams to various degrees [6,7]. Prevalent carbapenemases include KPC-2 (class A serine β-lactamase), OXA-48 (class D serine β-lactamase), and NDM (class B metallo-β-lactamase) [3,8]. Although carbapenems are to some extent inactivated by these enzymes combining two carbapenems may still result in synergistic interactions. Previous studies have hypothesized that the synergistic potential of such combinations mainly depends on the different enzymatic affinities among carbapenems. For example, ertapenem, with its relatively high affinity for carbapenemases, has been proposed to act as a suicide enzyme inhibitor that may restore the activity of a second carbapenem with lower enzymatic affinity [9,10,11,12].

While double-carbapenem treatment is sometimes referred to as a therapeutic option, clinical evidence is lacking [13,14,15,16,17,18,19]. Moreover, in vitro data on the synergistic effects are conflicting, and there is a lack of consensus on which carbapenems should be combined to best counteract the different enzymes [12,20,21,22,23,24]. The variable results between the studies may partly be due to strain-dependent factors. When using multidrug-resistant clinical isolates, other resistance mechanisms (e.g., the production of additional β-lactamases or porin alterations resulting in decreased drug permeability) are frequent and may influence antibiotic susceptibility as well as the ability of a combination to act synergistically.

In this study, we evaluated the activity of two-drug combinations of ertapenem, meropenem, and doripenem, at clinically achievable concentrations, against 51 genetically characterized clinical isolates of carbapenemase-producing *Escherichia coli* and *Klebsiella pneumoniae*. To assess the relative impact of different carbapenemases, we also included genetically modified *E. coli* strains in which genes encoding KPC-2, OXA-48, and NDM-1 were introduced into an otherwise identical genetic background. The activity of the single drugs and combinations was evaluated by 24 h automated time-lapse microscopy experiments [25,26,27] with a subsequent spot assay and in static time-kill experiments. Finally, we explored potential associations between phenotypic susceptibility to the tested antibiotics, carbapenemase genes, and the observed synergistic effects.

## 2. Results

### 2.1. Genetic Characterization and Antibiotic Susceptibility

Twenty-four clinical carbapenemase-producing *E. coli* isolates (4 KPC-2, 10 OXA-48, 4 NDM-1, and 6 NDM-5 producers) and 27 clinical carbapenemase-producing *K. pneumoniae* isolates (10 KPC-2, 7 OXA-48, 6 NDM-1, 1 NDM-5, and 3 NDM-1+OXA-48 producers) were included. Most isolates (46/51) harbored genes encoding additional β-lactamases, mainly CTX-M, TEM, OXA, and SHV (Table 1 and Table 2). In 20/51 strains, we found sequence variations (frameshift or premature stop codon) associated with the inactivation of the porin-encoding genes *ompF*, *ompK35,* and *ompK36*, which may affect β-lactam susceptibility. No loss of function mutations were identified in *ompC*. All clinical isolates were resistant to ertapenem, 30/51 were resistant to meropenem (10/24 *E. coli* and 20/27 *K. pneumoniae* isolates), and 33/51 were resistant to doripenem (11/24 *E. coli* and 22/27 *K. pneumoniae* isolates). The constructed NDM-1-producing *E. coli* strain was resistant to all three carbapenems, while the KPC-2 construct was resistant to ertapenem but susceptible to meropenem and doripenem. The construct producing OXA-48 was susceptible to all three carbapenems (Table 3).

### 2.2. Time-Lapse Microscopy Screening and Spot Assay with Clinical Isolates

**Figure 1:**
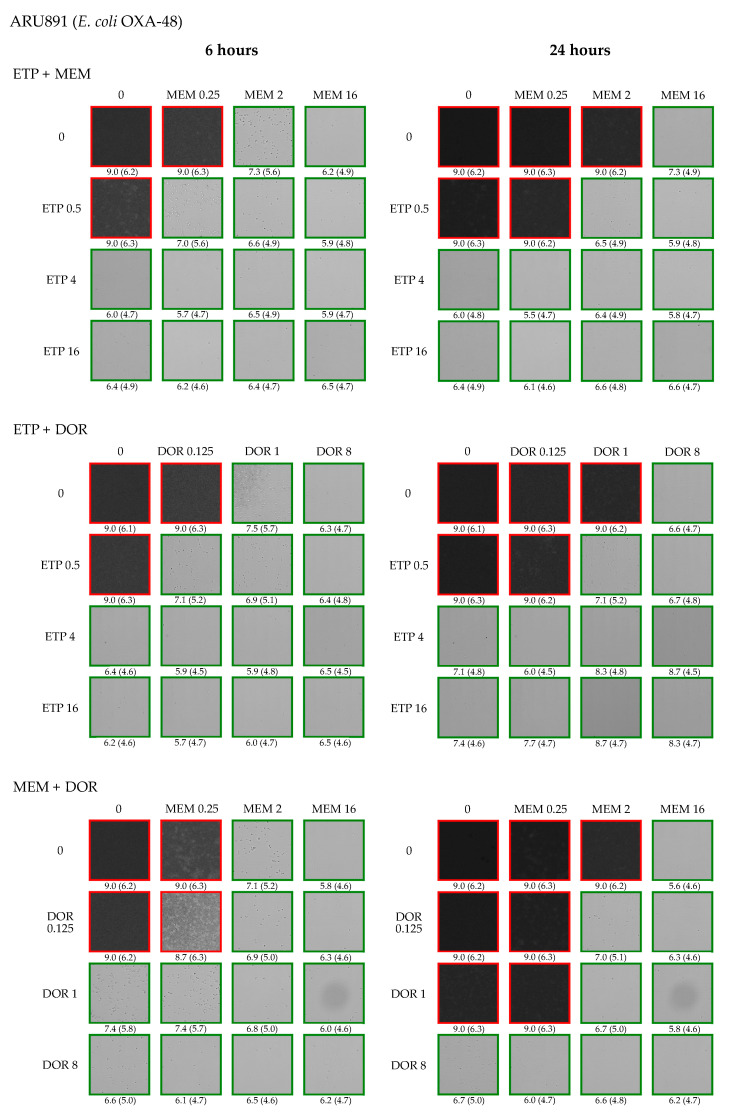
Example of output from time-lapse microscopy experiments. The images were obtained after 6 and 24 h of antibiotic exposure against OXA-48-producing *E. coli* (ARU891). Antibiotic concentrations are presented in mg/L. The BCA and SESA_max_ (in parentheses) values are presented below each image. If BCA and SESA_max_ exceed the predefined cut-off values (BCA > 8.0 and SESA_max_ > 5.8), indicating a bacterial density of approximately > 10^6^ CFU/mL, the image is marked with a red outline. If BCA and/or SESA_max_ are below the cut-off values, the image is marked with a green outline. Abbreviations: ETP—ertapenem; MEM—meropenem; DOR—doripenem.

For the clinical *E. coli* isolates, an enhanced effect in the time-lapse microscopy experiments was observed with the combination of ertapenem and meropenem against 8/24 isolates (4/4 KPC-2, 3/10 OXA-48, and 1/10 NDM producers) at 6 h and against 9/24 isolates (2/4 KPC-2, 7/10 OXA-48, and 0/10 NDM producers) at 24 h (Table 1, Figure 1, Supplementary Figure S1a). Ertapenem in combination with doripenem displayed an enhanced effect against 10/24 *E. coli* isolates (2/4 KPC-2, 8/10 OXA-48, and 0/10 NDM producers) at 6 h and against 9/24 *E. coli* isolates (2/4 KPC-2, 7/10 OXA-48, and 0/10 NDM producers) at 24 h (Table 1, Figure 1, Supplementary Figure S1b). Meropenem with doripenem displayed an enhanced effect against 11/24 *E. coli* isolates (1/4 KPC-2, 5/10 OXA-48, and 5/10 NDM producers) at 6 h and against 8/24 *E. coli* isolates (2/4 KPC-2, 6/10 OXA-48, and 0/10 NDM producers) at 24 h (Table 1, Figure 1, Supplementary Figure S1c).

For the clinical *K. pneumoniae* isolates, an enhanced effect of ertapenem and meropenem in combination was observed against 6/27 isolates (0/10 KPC-2, 2/7 OXA-48, 4/7 NDM and 0/3 NDM-1+OXA-48 producers) at 6 h and against 7/27 isolates (1/10 KPC-2, 4/7 OXA-48, 2/7 NDM and 0/3 NDM-1+OXA-48 producers) at 24 h (Table 2, Supplementary Figure S1a). Ertapenem in combination with doripenem displayed an enhanced effect against 7/27 *K. pneumoniae* isolates (2/10 KPC-2, 3/7 OXA-48, 2/7 NDM and 0/3 NDM-1+OXA-48 producers) at 6 h and against 3/17 *K. pneumoniae* isolates (1/10 KPC-2, 2/7 OXA-48, 0/7 NDM and 0/3 NDM-1+OXA-48 producers) at 24 h (Table 2, Supplementary Figure S1b). Meropenem and doripenem displayed an enhanced effect against 11/27 *K. pneumoniae* isolates (3/10 KPC-2, 2/7 OXA-48, 5/7 NDM, and 1/3 NDM-1+OXA-48 producers) at 6 h and against 8/27 *K. pneumoniae* isolates (0/10 KPC-2, 5/7 OXA-48, 2/7 NDM and 1/3 NDM-1+OXA-48 producers) at 24 h (Table 2, Supplementary Figure S1c).

In all cases where an enhanced effect was detected at 24 h in time-lapse microscopy experiments, a synergistic effect with the same carbapenem combination was also observed in the spot assay (Table 1 and Table 2, Supplementary Figure S1). In addition, the spot assay showed synergy in five cases where the time-lapse microscopy did not show an enhanced effect at 24 h. Most synergistic combinations also had a bactericidal effect: in 16/17 cases with ertapenem and meropenem, 13/14 cases with ertapenem and doripenem, and 16/18 cases with meropenem and doripenem.

A reduced effect of the combination, compared to the most active single drug, was detected at 6 h against four isolates: with ertapenem and meropenem against ARU713 (*E. coli* NDM-1), ARU714 (*E. coli* NDM-1), ARU731 (*K. pneumoniae* OXA-48) and ARU879 (*K. pneumoniae* NDM-1+OXA-48), and with meropenem and doripenem against ARU713 and ARU879 (Supplementary Figure S1a,c). Antagonism at 24 h, according to the spot assay, was observed in three cases: with ertapenem and meropenem against ARU874 (*K. pneumoniae* OXA-48) and ARU923 (*K. pneumoniae* NDM-1) and with meropenem and doripenem against ARU928 (*K. pneumoniae* NDM-5). Of note, synergistic and bactericidal effects were also detected for these combinations against the same strains when using other drug concentrations (Supplementary Figure S1a,c).

### 2.3. Associations between Antibiotic Susceptibility, Carbapenemase Genes and Synergy

Synergy rates were significantly higher in clinical *E. coli* isolates that were susceptible (S) or susceptible with increased exposure (I) to meropenem compared to resistant isolates with combinations of ertapenem and meropenem (*p* = 0.0006), ertapenem and doripenem (*p* = 0.0006), as well as meropenem and doripenem (*p* = 0.002) (Supplementary Table S1). Similarly, doripenem susceptibility (S or I) in *E. coli* was associated with synergy for combinations of ertapenem and meropenem (*p* = 0.0002), ertapenem and doripenem (*p* = 0.0045), and meropenem and doripenem (*p* = 0.0131). For *K. pneumoniae*, synergy with ertapenem and meropenem was associated with susceptibility (S or I) to meropenem (*p* = 0.0002) and doripenem (*p* = 0.0003). However, no association was found between antibiotic susceptibility and synergy with other combinations. All clinical isolates were resistant to ertapenem; consequently, the associations between susceptibility and synergy could not be assessed.

OXA-48 production was frequent among the isolates susceptible to meropenem (14/21 isolates) and doripenem (14/18 isolates) (Table 1 and Table 2). The presence of OXA-48 in *E. coli* and *K. pneumoniae* was associated with synergy for combinations of ertapenem and meropenem (*p* = 0.0027, *p* = 0.0496), ertapenem and doripenem (*p* = 0.0027, *p* = 0.0419), as well as meropenem and doripenem (*p* = 0.0104, *p* = 0.0235) when compared to non-OXA-48 (Supplementary Table S1). In contrast, metallo-β-lactamase production (NDM-1 or NDM-5) was frequent among isolates that were resistant to meropenem (18/30 isolates) and doripenem (20/33 isolates) (Table 1 and Table 2). The presence of NDM in *E. coli* (10/24 isolates) was associated with lower synergy rates with ertapenem and meropenem (*p* = 0.0006), ertapenem and doripenem (*p* = 0.0006), and meropenem and doripenem (*p* = 0.002) (Supplementary Table S1).

### 2.4. Time-Kill Experiments with Clinical Isolates

Ten clinical isolates against which at least one combination exhibited synergy and a bactericidal effect in the spot assay were evaluated in static time-kill experiments (Table 4). Synergy at 2, 6, or 24 h with at least one of the combinations was observed against 4/4 OXA-48-producing isolates (2/2 *E. coli* and 2/2 *K. pneumoniae*) and 1/3 KPC-2-producing isolates (1/2 *E. coli* and 0/1 *K. pneumoniae*). No synergistic effect was detected against the three NDM-producing *K. pneumoniae* isolates.

### 2.5. Time-Kill Experiments with Constructed and Wild-Type E. coli

**Figure 2:**
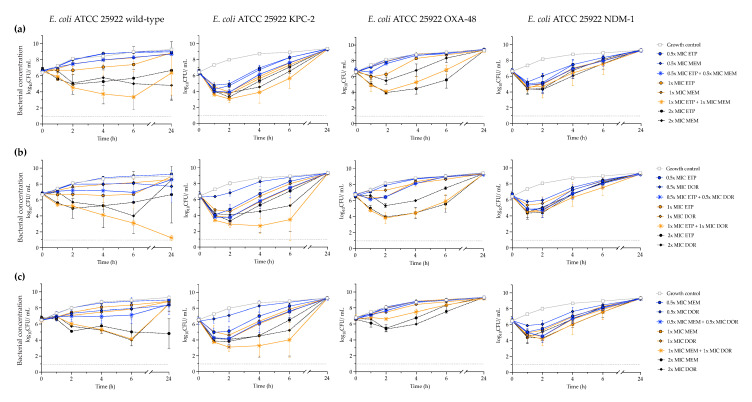
Mean bacterial concentrations during 24 h time-kill experiments with ertapenem, meropenem, and doripenem, alone and in two-drug combinations against *E. coli* ATCC 25922 wild-type and constructed carbapenemase-producing strains. (**a**) Ertapenem and meropenem; (**b**) Ertapenem and doripenem; (**c**) Meropenem and doripenem. The lower limit of detection (dotted line) was 1 log_10_ CFU/mL. Abbreviations: ETP—ertapenem; MEM—meropenem; DOR—doripenem.

The constructed KPC-2-, OXA-48-, and NDM-1-producing *E. coli* strains were exposed to ertapenem, meropenem, and doripenem alone at concentrations of 0.5 ×, 1 × and 2 × MIC and in two-drug combinations at 0.5 × MIC, and 1 × MIC (Figure 2). Synergy was not found with any combination at concentrations of 0.5 × MIC, and the bacterial killing effect was generally similar to that of the single drugs at 1 × MIC. At concentrations of 1 × MIC, synergy was observed at early time points against the KPC-2- and OXA-48-producing strains. However, due to regrowth, no synergy was detected at 24 h. The ertapenem and doripenem combination, as well as meropenem and doripenem in combination, showed synergy against the KPC-2-producing strain at 4 and 6 h (Figure 2b,c). At these time points, the two-drug combinations were superior (1.2–3.6 log_10_ CFU/mL reductions) to the single drugs at 2 × MIC. Ertapenem and meropenem showed synergy against the OXA-48-producing strain at 2 and 4 h, and the combination of ertapenem and doripenem was synergistic at 2, 4, and 6 h (Figure 2a,b). However, bacterial killing was similar (<1 log_10_ difference in CFU/mL) to that of the most effective single drug at 2 × MIC. No synergistic activity was observed against the NDM-1-producing construct. All three double-carbapenem combinations at concentrations of 1× MIC exhibited synergistic effects against the wild-type *E. coli* (ATCC 25922) strain in at least two consecutive time points. When synergy was observed, the bacterial killing was superior (≥1 log_10_ CFU/mL reduction) to the most effective single antibiotic at 2 × MIC in 4/9 cases (Figure 2).

To assess the possible emergence of resistance in the regrowing populations during time-kill experiments, 24 h samples were spread on plates containing ertapenem, meropenem, or doripenem at 4 × and 8 × MIC. Resistance development was rare, and mutants were obtained only in five experiments at 4 × MIC (Supplementary Table S2). MIC increased in the isolated mutants ranging from 4- to 64-fold for ertapenem, 2- to 32-fold for meropenem, and 2- to 32-fold for doripenem. Four of the five mutants displayed a decrease in growth rate ranging from 6 to 44% compared to the parental strain. Whole-genome sequencing did not reveal any sequence variations previously known to be associated with decreased carbapenem susceptibility, such as amino acid substitutions or gene amplifications of β-lactamase genes [3], or mutations in porin-encoding genes, penicillin-binding proteins, and other cell wall-associated genes (Supplementary Table S3).

## 3. Discussion

In this study, we evaluated the activity of ertapenem, meropenem, and doripenem combinations against carbapenemase-producing *Enterobacterales*. Whole-genome sequencing revealed that most *E. coli* and all *K. pneumoniae* clinical isolates harbored additional β-lactamases. Mutations likely to cause porin alterations were frequent in *K. pneumoniae* and were also found in some of the *E. coli* isolates. To compare the effects of double-carbapenem combinations in the presence of only a single carbapenemase, we also used genetically modified *E. coli* strains producing KPC-2, OXA-48, or NDM-1 in an otherwise isogenic background. Enhanced and synergistic effects of the combinations were frequently found against OXA-48-producing isolates, whereas the efficacy of the combinations was low against KPC-2 and negligible against NDM producers.

In *E. coli*, OXA-48 production was associated with synergy. By contrast, we did not find synergy against any of the NDM-producing clinical *E. coli* isolates or the constructed NDM-1 strain. Statistical analysis of the association between KPC-2 production and synergy was not applicable due to the small sample size; however, synergy with at least one of the combinations was observed against 3/4 isolates. OXA-48 production was also associated with synergy in *K. pneumoniae*. Although no statistically significant association was found, synergy rates were low in *K. pneumoniae* isolates producing KPC-2 (2/10) and NDM (3/10). Time-kill experiments with clinical isolates showed a similar trend; synergy was observed against the 4/4 OXA-48-, 1/3 KPC-2-, and 0/3 NDM-producing isolates. In time-kill experiments using constructed *E. coli* strains, synergistic effects were observed at early time points against the OXA-48- and KPC-2-producing strains, whereas no synergy was detected against the NDM-1 producer.

The poor activity of double-carbapenem combinations against NDM-producing isolates in this study is in line with previous reports [21,22]. This association is probably attributed mainly to the highly efficient inactivation of carbapenems by NDM [28]. In contrast, OXA-48 exhibits poor hydrolytic activity compared to KPC-2, and especially NDM-1 [6], as is also reflected in the high susceptibility rates for carbapenems in OXA-48-producing isolates. Most previous studies that evaluated double-carbapenem treatment included mainly KPC-producing *K. pneumoniae*, whereas data on *K. pneumoniae* producing other carbapenemases and *E. coli* are scarce. Our findings highlight the need to consider more specific genotype-phenotype associations when evaluating combination effects.

As expected, due to the differences in enzymatic activity, associations were also revealed between carbapenem susceptibility and synergy. With all three combinations, synergy rates were higher in *E. coli* isolates susceptible to meropenem and doripenem compared to the resistant isolates. For *K. pneumoniae*, the synergy rate with ertapenem and meropenem was higher in meropenem- and doripenem-susceptible isolates. However, in *K. pneumoniae*, no statistically significant association was detected for the other combinations. We hypothesize that the difference in results between *E. coli* and *K. pneumoniae* in this regard may be due to the lower susceptibility rates in *K. pneumoniae*.

Thus, our observations suggest that the ability of double-carbapenem combinations to achieve synergy at clinically achievable concentrations is at least partly dependent on the susceptibility to the constituent antibiotics. In our study, we did not explore the synergistic potential of drug concentrations that exceeded the maximum free drug concentrations in the patient’s plasma. Considering this, the probability of synergy is expected to be lower against isolates with high MICs. A similar association was reported in another study in which the degree of synergy was higher in *K. pneumoniae* isolates with lower meropenem MICs (range up to 128 mg/L) [10]. In contrast, another study reported that synergy with double-carbapenem combinations in checkerboards was more likely to occur in clinical isolates showing higher MICs [21]. The discrepancy in results between studies may be due to methodological differences, particularly the range of drug concentrations tested, which determines the probability of detecting synergy with the combinations.

To our knowledge, double-carbapenem therapy was first suggested by Bulik et al., who reported an enhanced activity of ertapenem and doripenem against KPC-3-producing *K. pneumoniae* in dynamic in vitro experiments and an in vivo murine thigh infection model [9]. Ertapenem has been the most frequently used carbapenem in double-carbapenem regimens [13], and ertapenem-containing combinations have been reported to be more effective than other double-carbapenem regimens [9,11,12,17,29]. This finding has been attributed to ertapenem’s high enzymatic affinity, allowing it to efficiently occupy the carbapenemase and prevent the degradation of the second drug [13,14,30]. However, some studies also reported indifference with ertapenem combinations [21,23]. In one study using recombinant KPC-2-, OXA-48-, and NDM-1-producing *E. coli* strains, synergy in the checkerboards were identified only against the KPC-2-producing strain and only with imipenem combinations [21]. In our study, the synergy rates were similar for ertapenem combinations compared to meropenem and doripenem, and an enhanced activity at 6 h was more frequently observed with meropenem and doripenem. Hence, our data differ from some of the previous studies and do not support the assumption that ertapenem is generally the preferred carbapenem.

Considering the proposed mechanism of synergistic interaction, i.e., the competitive inhibition of the carbapenemase, we wanted to explore whether the perceived synergy rather reflected an additive effect that might also be achieved by increasing the concentration of a single carbapenem. Therefore, in the time-kill experiments with wild-type and constructed *E. coli* strains, we compared the activity of the combination to both single drugs at 2-fold higher drug concentrations. In 10/18 cases, where synergy was observed, the antibacterial activity at that specific time point was similar to one or both single drugs at a 2-fold higher concentration. In those cases, using a single carbapenem at a higher dose would probably be equally effective as using the combination. However, in the remaining 8/18 cases the activity of the combination was superior to the single drugs at 2-fold higher concentrations. Of note, the degree of synergy was unexpectedly higher against wild-type *E. coli* ATCC 25922 than against the constructs. These findings suggest there may be additional mechanisms of synergistic interactions between the carbapenems, e.g., due to their different affinities for penicillin-binding proteins [31,32], which are not related to the presence of carbapenemases.

Regrowth at 24 h was frequent in the time-kill experiments and is commonly reported in studies with β-lactam antibiotics [10,26,31,33,34]. This phenomenon can be caused, for example, by the degradation of the antibiotic during experiments, the emergence of resistant mutants, or tolerance. We believe that drug degradation was the main reason for regrowth in our study. Carbapenems generally exhibit poor stability in solution, and the presence of carbapenemases further accelerates the reduction in antibiotic concentration over time [33,35,36]. Population analysis profiling revealed that the emergence of resistant mutants was rare in experiments with the constructed strains. Although mutants with reduced carbapenem susceptibility were isolated, most of which showed decreased growth rates, we could not identify any sequence variations known to cause reduced susceptibility to carbapenems.

A large number of isolates, genetic characterization, and systematic approaches using several in vitro methods are strengths of this study. The agreement was high between the automatic and manual readouts in the time-lapse microscopy experiments; combinations that showed an enhanced activity according to BCA and SESA_max_ were invariably synergistic in the spot assay. The influence of gene expression levels and efflux systems were not assessed in this study but should be considered in future research as these factors influence the susceptibility to carbapenems [3,37,38]. We acknowledge that in vitro findings cannot be directly translated to a clinical situation because of differences in growth conditions, bacterial inocula, drug concentrations, and the lack of immune system effects. For example, poor in vivo and clinical outcomes have been reported for carbapenem treatment against OXA-48-producing strains, although these bacteria are frequently determined susceptible in vitro [39].

In conclusion, our results suggest that the benefit of double-carbapenem therapy against carbapenemase-producing *Enterobacterales* is limited. Synergy was frequently demonstrated against OXA-48-producing *E. coli* and *K. pneumoniae*. Lower activity was found against KPC-2 and especially NDM producers and against isolates that were phenotypically resistant to meropenem and doripenem. Animal and clinical studies are warranted to validate our in vitro findings. Further investigation of genotype-phenotype associations may provide insights into the therapeutic potential and limitations of antibiotic combinations against strains with different setups of resistance genes.

## 4. Materials and Methods

### 4.1. Strains, Growth Conditions and Antibiotics

The clinical isolates were obtained from the Public Health Agency of Sweden. All strains were grown at 37 °C with cation-adjusted Mueller-Hinton (MH-II) (BD Diagnostics, Sparks, MD, USA) broth or MH-II agar unless stated otherwise. Viable counts were read after 24 h. Antibiotics were purchased from Sigma-Aldrich (Merck KGaA, Darmstadt, Germany) and prepared according to the manufacturer’s instructions.

### 4.2. Strain Construction

Genetic modifications were performed in *E. coli* ATCC 25922 (ARU961) carrying the pSIM5-*tet* plasmid (from DA27235; *E. coli* MG1655 pSIM5-*tet*), which encodes the λ-red-recombineering system. Strains carrying pSIM5-*tet* were grown at 30 °C, and the λ-red-recombineering system was induced by incubating cells at 42 °C for 15 min. A de-salted purified PCR product (Thermo Scientific™ GeneJET™ Gel Extraction Kit) of a *cat-sacB* cassette (from DA46472; *E. coli* MG1655 *∆bglGFB:cat-sacB* pSIM6) with flanking transcriptional terminators (see Supplementary Table S4 for primers) was electroporated (Gene Pulser Xcell system, Bio-Rad™ (Hercules, CA, USA), 2.5 kV, 25 mF, and 200 W) into the induced electrocompetent *E. coli* ATCC 25922 pSIM5-*tet* transformants. The cassette was integrated into the *bgl* operon on the chromosome using λ-red recombination, and transformants were selected on MH-II agar containing 12 μg/mL chloramphenicol. The *cat-sacB* cassette was subsequently exchanged for a carbapenemase gene with its native promoter sequence by λ-red recombination with a purified PCR product of *bla*_OXA-48_, *bla*_KPC-2,_ or *bla*_NDM-1_ (see Supplementary Table S4 for primers) but the flanking transcriptional terminators were left intact. Transformants were selected on MH-II agar supplemented with 5% sucrose, which counter-selects for the *cat-sacB* cassette. Due to a low integration frequency, transformants with *bla*_KPC-2_ were selected on 100 μg/mL ampicillin. The temperature-sensitive pSIM5-*tet* plasmid was removed by growing constructed strains at 37 °C. Successful integrations of carbapenemase genes were verified with PCR and local Sanger sequencing (Eurofins Genomics, Aarhus, Denmark).

### 4.3. Antibiotic Susceptibility Testing

The MICs of ertapenem, meropenem, and doripenem were determined using broth microdilution with *E. coli* ATCC 25922 as a quality control, according to EUCAST guidelines [40,41]. The strains were categorized as susceptible (S), susceptible with increased exposure (I), or resistant (R), according to EUCAST clinical breakpoints, version 12.0 [42].

### 4.4. Screening Using Time-Lapse Microscopy

Automated time-lapse microscopy (oCelloScope, BioSense Solutions ApS, Farum, Denmark) was used as previously described [25,26,27] to screen the activity of ertapenem, meropenem, and doripenem both alone and in two-drug combinations against 51 KPC-2-, NDM- and OXA-48-producing clinical *E. coli* and *K. pneumoniae* isolates. *Pseudomonas aeruginosa* ATCC 27853 was used as a quality control. Clinically achievable free (non-protein bound) antibiotic concentrations were used, with concentration ranges adapted to the MIC values of the tested isolates. Ertapenem was added to concentrations of 0.5, 4, and 16 mg/L. Meropenem was added to 2, 16, and 64 mg/L or 0.25, 2, and 16 mg/L. Doripenem was added to 1, 8, and 32 mg/L or 0.125, 1, and 8 mg/L.

The starting inoculum was adjusted to ~10^6^ CFU/mL. The experiments were performed in flat-bottomed 96-well microtiter plates (Greiner Bio-One GmbH, Frickenhausen, Germany). The oCelloScope instrument was kept at 37 °C and set to generate images of each well every 15 min for 24 h. The bacterial density at 24 h was determined by using the UniExplorer software version 6.0.0 (Philips BioCell A/S, Allerød, Denmark) to calculate the background-corrected absorption (BCA) and segmentation extracted surface area (SESA). BCA > 8.0 and maximum SESA (SESA_max_) > 5.8 were used as cut-off values to indicate a bacterial density of approximately >10^6^ CFU/mL. If the bacterial density was below at least one of the cut-off values with a combination but not with either of the constituent single antibiotics, the combination was considered to have an enhanced effect. A combination was considered to exhibit a reduced effect if both BCA and SESA_max_ were above the cut-offs with the combination, while one or both of BCA and SESA_max_ were below the cut-offs with one or both single antibiotics [26,27].

### 4.5. Spot Assay

Directly following the 24-hour time-lapse microscopy experiments, a spot assay was performed to provide more detailed information on the bacterial concentrations. A volume of 10 μL of undiluted and serially diluted samples from the microplate wells was spotted on the agar plates. Following overnight incubation at 37 °C, the viable count (log_10_ CFU/mL) was determined. The LOD was 2 log_10_ CFU/mL; no visible growth was, therefore, noted as 1 log_10_ CFU/mL to not overestimate the effect.

### 4.6. Time-Kill Experiments

The starting cultures were prepared by diluting an overnight culture 100-fold in a pre-warmed broth to achieve a starting inoculum of ~10^6^ CFU/mL. The activity of ertapenem, meropenem and doripenem was tested alone and in two-drug combinations. For the clinical isolates, we used the drug concentrations at which a combination had exhibited synergy in the spot assay. If no synergy was detected, the highest drug concentration at which growth occurred was used. Samples were taken at 0, 2, 6, and 24 h, were serially diluted, and 100 μL was spread on agar plates. The constructed strains and the wild-type parental strain were exposed to concentrations of 0.5 × and 1 × MIC for each carbapenem alone and in two-drug combinations. In addition, 2 × MIC of the single drugs was tested to compare the activities of two carbapenems at 1 × MIC and one carbapenem at 2 × MIC. Samples were taken at 0, 1, 2, 4, 6, and 24 h. Experiments were performed in at least two biological replicates, and the mean values (log_10_ CFU/mL) were used in the analysis.

### 4.7. Definitions of Synergy, Antagonism and Bactericidal Effect

In the spot assay and time-kill experiments, synergy was defined as a ≥2 log_10_ decrease in CFU/mL with the combination compared to the most effective single antibiotic. Antagonism was defined as a ≥2 log_10_ higher CFU/mL with the combination compared to the most effective single antibiotics. A bactericidal effect was defined as a ≥3 log_10_ reduction in CFU/mL compared to the starting inoculum [43].

### 4.8. Resistance Development

The resistance development during time-kill experiments was evaluated in one replicate for each constructed *E. coli* strain and the wild-type parental strain. A volume of 100 μL of undiluted 24-hour samples and a 10-fold dilution was spread on agar plates, each containing a carbapenem at concentrations of 4 × MIC and 8 × MIC. The mutant frequency was calculated as (*r/Nt*), where *r* is the number of mutants (CFU/mL) from the selective plate and *Nt* is the total number of viable cells from non-selective plates (CFU/mL).

### 4.9. Growth Rate Measurements

Growth rates were measured using a Bioscreen C MBR spectrophotometer (Oy Growth Curves Ab Ltd.). Overnight cultures were diluted 1000-fold in broth, and 300 µL were transferred to the honeycomb plates (Oy Growth Curves Ab Ltd., Turku, Finland). Three biological replicates were included, as well as one technical replicate for each biological replicate. Bacterial growth was measured at 37 °C with shaking by optical density (OD_600_) every 4 min for 24 h. The plotting of bacterial growth curves and calculation of growth rates were performed using BAT 2.0 (Bioscreen Analysis Tool) [44]. For OD_600,_ values between 0.02 and 0.1 growth were considered exponential, and the growth rate was defined as the slope of the curve during exponential growth.

### 4.10. Whole Genome Sequencing and Genetic Characterization

The sequencing of clinical isolates was performed by the Public Health Agency of Sweden using IonTorrent S5 XL. The ResFinder tool (CLC Microbial Genomics Module 22.1, CLC Genomics Workbench 22.0.2, CLCbio, Qiagen, Aarhus, Denmark) was used to identify β-lactamase genes. Sequence variations in β-lactamase genes and porin genes were evaluated in CLC Main Workbench version 21 (CLCbio, Qiagen).

Reference genes from the ResFinder database were used to identify amino acid sequence variations in genes encoding β-lactamases. The loss of function mutations (premature stop codons and frameshifts) in genes expressing porins were identified. We did not report other sequence variations in porin genes due to their large natural variation and uncertain biological function. An in-house reference sequence for *E. coli* MG1655 K-12 was used as a reference for alignments with *ompC* and *ompF*, while *K. pneumoniae* ATCC 35657 (NCBI Reference Sequence NZ_CP015134) was used as a reference for alignments with *ompK35* and *ompK36*. The whole-genome sequencing of mutants with a decreased susceptibility to carbapenems following time-kill experiments with constructed strains was performed using Illumina MiSeq (Illumina Inc., San Diego, CA, USA). Genomic DNA was prepared using the Epicentre MasterPure^TM^ DNA purification Kit (Illumina Inc.) according to the manufacturer’s instructions. Sequences were assembled against reference *E. coli* ATCC 25922 (NZ_CP009072, NZ_CP009073, NZ_CP009074) in CLC Genomics Workbench version 21 (CLCbio, Qiagen) and analyzed for genetic variations (SNPs, InDels) in CLC Main Workbench version 21 (CLCbio, Qiagen).

### 4.11. Statistical Analyses

Fisher’s Exact Test was performed using GraphPad Prism (version 9.4.0) to test for associations between synergistic effects in the spot assay and susceptibility to the tested carbapenems or the presence of specific carbapenemase genes. Associations with *p* < 0.05 were regarded as statistically significant.

## Figures and Tables

**Table 1:** Summary of results from time-lapse microscopy experiments (6 and 24 h) and spot assay (24 h) for double-carbapenem combinations against clinical carbapenemase-producing *E. coli*. MIC values for ertapenem, meropenem, and doripenem are classified according to EUCAST clinical breakpoints, version 12.0. Amino acid changes in β-lactamases are written in parentheses. Detected loss of function mutations in porin-encoding genes (OmpC/F) are presented. Carbapenem combinations showing an enhanced effect in the time-lapse microscopy experiments or synergy in the spot assay are highlighted in orange. Synergistic combinations that also showed a bactericidal effect are marked with a thick outline.

*E. coli* Strain	Carbapenemase	Other β-Lactamases	OmpC	OmpF	MIC (mg/L)			ETP + MEM			ETP + DOR			MEM + DOR		
					ETP	MEM	DOR	Time-lapse 6 h	Time-lapse 24 h	Spot Assay	Time-lapse 6 h	Time-lapse 24 h	Spot Assay	Time-lapse 6 h	Time-lapse 24 h	Spot Assay
ARU887	KPC-2	-			2 (R)	0.25 (S)	0.25 (S)									
ARU888	KPC-2	-			8 (R)	2 (S)	1 (S)									
ARU894	KPC-2	TEM-1A, OXA-9 (W112 *)			16 (R)	2 (S)	1 (S)									
ARU1141	KPC-2	CTX-M-15, TEM-1B			32 (R)	8 (I)	4 (R)									
ARU716	OXA-48	CTX-M-14			8 (R)	2 (S)	1 (S)									
ARU722	OXA-48	CTX-M-15, OXA-1		Y254fs N259 *	16 (R)	2 (S)	2 (I)									
ARU889	OXA-48	-			4 (R)	1 (S)	1 (S)									
ARU890	OXA-48	-			4 (R)	1 (S)	1 (S)									
ARU891	OXA-48	TEM-1B			2 (R)	0.5 (S)	1 (S)									
ARU896	OXA-48	CTX-M-15			4 (R)	0.5 (S)	0.25 (S)									
ARU898	OXA-48	CTX-M-15			8 (R)	0.5 (S)	0.5 (S)									
ARU903	OXA-48	CTX-M-15, TEM-1B, OXA-1			2 (R)	0.5 (S)	0.25 (S)									
ARU991	OXA-48	TEM-1B			4 (R)	1 (S)	2 (I)									
ARU992	OXA-48	CTX-M-14			8 (R)	2 (S)	2 (I)									
ARU711	NDM-1	CTX-M-27			32 (R)	32 (R)	32 (R)									
ARU713	NDM-1	CTX-M-27			>32 (R)	32 (R)	>32 (R)									
ARU714	NDM-1	CTX-M-27			>32 (R)	32 (R)	32 (R)									
ARU892	NDM-1	-			32 (R)	32 (R)	16 (R)									
ARU709	NDM-5	CTX-M-15, TEM-1B, CMY-2, OXA-1			>32 (R)	32 (R)	32 (R)									
ARU717	NDM-5	TEM-1B			>32 (R)	32 (R)	32 (R)									
ARU910	NDM-5	CMY-42		Y254fs N259 *	>32 (R)	>32 (R)	>32 (R)									
ARU912	NDM-5	CTX-M-15, TEM-1B, OXA-1		Y254fs N259 *	>32 (R)	>32 (R)	>32 (R)									
ARU913	NDM-5	TEM-1B			32 (R)	32 (R)	16 (R)									
ARU917	NDM-5	CTX-M-15, TEM-1B, OXA-1		Y254fs N259 *	>32 (R)	>32 (R)	32 (R)									

Abbreviations: ETP, ertapenem; MEM, meropenem; DOR, doripenem; S, susceptible; I, susceptible increased exposure; R, resistant; *, premature stop codon; fs, frameshift.

**Table 2:** Summary of results from time-lapse microscopy experiments (6 and 24 h) and spot assay (24 h) for double-carbapenem combinations against clinical carbapenemase-producing *K. pneumoniae*. MIC values for ertapenem, meropenem, and doripenem are classified according to EUCAST clinical breakpoints, version 12.0. Amino acid changes in β-lactamases are written in parentheses. Detected loss of function mutations in porin-encoding genes (OmpK36/35) are presented. Carbapenem combinations showing an enhanced effect in the time-lapse microscopy experiments or synergy in the spot assay are highlighted in orange. Synergistic combinations that also showed a bactericidal effect are marked with a thick outline.

*K. pneumoniae* Strain	Carbapenemase	Other β-Lactamases	OmpK36	OmpK35	MIC (mg/L)			ETP + MEM			ETP + DOR			MEM + DOR		
					ETP	MEM	DOR	Time-lapse 6 h	Time-lapse 24 h	Spot Assay	Time-lapse 6 h	Time-lapse 24 h	Spot Assay	Time-lapse 6 h	Time-lapse 24 h	Spot Assay
ARU737	KPC-2	SHV-187 (K3M, L33Q)		E312 *	>32 (R)	>32 (R)	>32 (R)									
ARU869	KPC-2	TEM-1A (S128fs), OXA-9 (W112 *), SHV-187 (K3M, L33Q)		E42fs G111 *	>32 (R)	>32 (R)	>32 (R)									
ARU871	KPC-2	CTX-M-15, TEM-1B, CMY-2, OXA-9 (W112 *), OXA-10, SHV-187 (K3M, L33Q)		E42fs G111 *	>32 (R)	>32 (R)	>32 (R)									
ARU919	KPC-2	CTX-M-65, TEM-1B, SHV-12		N29fs I69 *	>32 (R)	>32 (R)	>32 (R)									
ARU920	KPC-2	TEM-1A, OXA-9 (W112*), SHV-12		E42fs G111 *	>32 (R)	>32 (R)	>32 (R)									
ARU1011	KPC-2	CTX-M-15, SHV-28			>32 (R)	32 (R)	16 (R)									
ARU1015	KPC-2	TEM-1A, OXA-9 (M1Del, W112 *), SHV-12		E42fs G111 *	>32 (R)	>32 (R)	>32 (R)									
ARU1016	KPC-2	TEM-1A, OXA-9 (W112 *), SHV-187 (K3M, L33Q)		E42fs G111*	>32 (R)	>32 (R)	>32 (R)									
ARU1019	KPC-2	TEM-1A, OXA-9 (W112 *), SHV-187 (K3M)		L63 *	>32 (R)	>32 (R)	>32 (R)									
ARU1144	KPC-2	CTX-M-15, TEM-1B, OXA-1, SHV-187 (K3M, L33Q)			4 (R)	1 (S)	1 (S)									
ARU731	OXA-48	CTX-M-15, TEM-1A, OXA-1, OXA-9, SHV-187 (K3M)		G62fs L63 *	>32 (R)	32 (R)	32 (R)									
ARU734	OXA-48	CMY-4, SHV-187 (K3M, L33Q)			>32 (R)	>32 (R)	32 (R)									
ARU735	OXA-48	CMY-4, SHV-187 (K3M, L33Q)			4 (R)	1 (S)	1 (S)									
ARU736	OXA-48	CMY-4, SHV-187 (K3M, L33Q)	L32 *		>32 (R)	32 (R)	32 (R)									
ARU873	OXA-48	CTX-M-15, TEM-1B, OXA-1, SHV-11			16 (R)	2 (S)	2 (I)									
ARU874	OXA-48	CTX-M-15, TEM-1B, OXA-1, SHV-11			8 (R)	2 (S)	2 (I)									
ARU1005	OXA-48	CTX-M-15, TEM-1B, OXA-1, SHV-28			8 (R)	2 (S)	2 (I)									
ARU601	NDM-1	CTX-M-15, TEM-1B, OXA-1, OXA-9, CMY-4, SHV-187 (K3M, L33Q)			>32 (R)	>32 (R)	>32 (R)									
ARU725	NDM-1	CTX-M-15, TEM-1B, SHV-12	T123fs Q172 *		>32 (R)	>32 (R)	>32 (R)									
ARU726	NDM-1	CTX-M-15, SHV-11		D84fs L103 *	>32 (R)	16 (R)	32 (R)									
ARU884	NDM-1	CTX-M-15, TEM-1B, OXA-1, OKP-A-8			32 (R)	8 (I)	8 (R)									
ARU923	NDM-1	CTX-M-15, OXA-1, SHV-187 (K3M, L33Q)			>32 (R)	8 (I)	16 (R)									
ARU733	NDM-1	TEM-1B, OXA-1, SHV-187 (K3M, L33Q)			>32 (R)	>32 (R)	>32 (R)									
ARU928	NDM-5	CTX-M-15, TEM-1B, OXA-1, SHV-187 (K3M, L33Q)		K226fs E256 *	>32 (R)	32 (R)	>32 (R)									
ARU724	NDM-1 + OXA-48	CTX-M-15, TEM-1B, OXA-1, SHV-11		K3fs Ins29 *	>32 (R)	16 (R)	32 (R)									
ARU879	NDM-1 + OXA-48	CTX-M-15, TEM-1B, OXA-1, SHV-28		N240fs E256 *	>32 (R)	>32 (R)	>32 (R)									
ARU882	NDM-1 + OXA-48	CTX-M-15, SHV-28		N240fs E256 *	>32 (R)	>32 (R)	>32 (R)									

Abbreviations: ETP, ertapenem; MEM, meropenem; DOR, doripenem; S, susceptible; I, susceptible increased exposure; R, resistant; *, premature stop codon; fs, frameshift.

**Table 3:** MIC values for ertapenem, meropenem, and doripenem for *E. coli* ATCC 25922 and constructed carbapenemase-producing strains. MICs are classified according to EUCAST clinical breakpoints, version 12.0.

Strain	Genotype	MIC (mg/L)		
		ETP	MEM	DOR
ARU961	ATCC 25922 wild-type	0.0078 (S)	0.016 (S)	0.031 (S)
ARU1026	ATCC 25922 *bglG/F/B*::*bla*_KPC-2_	4 (R)	2 (S)	1 (S)
ARU1027	ATCC 25922 *bglG/F/B*::*bla*_NDM-1_	16 (R)	16 (R)	16 (R)
ARU1028	ATCC 25922 *bglG/F/B*::*bla*_OXA-48_	0.125 (S)	0.031 (S)	0.062 (S)

Abbreviations: ETP, ertapenem; MEM, meropenem; DOR, doripenem; S, susceptible; R, resistant.

**Table 4:** Mean bacterial concentrations (log_10_ CFU/mL) at 0, 2, 6, and 24 h during time-kill experiments with ertapenem, meropenem, and doripenem, alone and in two-drug combinations against clinical carbapenemase-producing isolates. The standard deviation (SD) at each time point is shown. Synergy (≥2 log_10_ reduction in CFU/mL compared to the most effective single antibiotic) is highlighted in orange. Bactericidal effects (≥3 log_10_ reduction in CFU/mL compared to the starting inoculum) are highlighted in green.

Strain	Antibiotic Concentrations (mg/L)	0 h	2 h			6 h			24 h		
		log_10_ CFU/mL ± SD	log_10_ CFU/mL ± SD	Δ ^a^	Δ ^b^	log_10_ CFU/mL ± SD	Δ^a^	Δ^b^	log_10_ CFU/mL ± SD	Δ ^a^	Δ ^b^
*E. coli* KPC-2 (ARU888)	Growth control	6.87 ± 0.16	8.36 ± 0.07		1.49	9.18 ± 0.21		2.31	9.43 ± 0.00		2.56
	ETP 16	6.75 ± 0.22	3.10 ± 0.43		−3.65	5.34 ± 0.08		−1.41	9.35 ± 0.01		2.6
	MEM 16	6.88 ± 0.25	2.92 ± 0.61		−3.96	3.88 ± 2.28		−3	9.35 ± 0.16		2.47
	DOR 8	6.92 ± 0.06	2.71 ± 0.04		−4.21	2.80 ± 1.20		−4.12	6.99 ± 3.13		0.07
	ETP 16 +MEM 16	6.88 ± 0.16	2.30 ± 0.16	−0.63	−4.58	1.94 ± 0.48	−1.94	−4.94	9.04 ± 0.35	−0.31	2.16
	ETP 16 + DOR 8	6.86 ± 0.20	3.31 ± 1.64	0.6	−3.55	2.40 ± 0.24	−0.4	−4.46	5.74 ± 3.95	−1.25	−1.12
	MEM 16 + DOR 8	6.84 ± 0.26	2.19 ± 0.16	−0.52	−4.65	4.24 ± 2.77	1.45	−2.6	5.04 ± 0.62	−1.96	−1.80
*E. coli* KPC-2 (ARU1141)	Growth control	6.19 ± 0.1	7.99 ± 0.13		1.8	9.23 ± 0.1		−3.04	9.10 ± 0.01		2.91
	ETP 16	6.17 ± 0.19	3.87 ± 1.62		−2.3	6.18 ± 1.61		−0.01	9.17 ± 0.01		3.00
	MEM 16	6.23 ± 0.11	1.99 ± 0.97		−4.24	1.35 ± 0.49		−4.88	4.51 ± 4.96		−1.72
	MEM 64	6.25 ± 0.16	1.30 ± 0.42		−4.95	1.45 ± 0.64		−4.8	3.80 ± 1.82		−2.45
	DOR 8	6.22 ± 0.11	3.87 ± 0.03		−2.35	1.96 ± 1.36		−4.26	7.12 ± 2.73		0.90
	ETP 16 + MEM 64	6.27 ± 0.11	2.05 ± 1.48	0.75	−4.22	3.01 ± 2.84	1.56	−3.26	4.08 ± 2.83	0.28	−2.19
	ETP 16 + DOR 8	6.16 ± 0.12	1.91 ± 0.86	−1.96	−4.25	2.08 ± 1.53	0.12	−4.08	2.05 ± 1.48	−5.07	−4.11
	MEM 16 + DOR 8	6.18 ± 0.11	2.46 ± 1.07	0.47	−3.72	1.00 ± 0.00	−0.35	−5.18	2.05 ± 1.48	−2.47	−4.13
*E. coli* OXA-48 (ARU891)	Growth control	6.80 ± 0.13	8.32 ± 0.06		1.52	8.97 ± 0.09		2.17	9.42 ± 0.07		2.62
	ETP 0.5	6.79 ± 0.07	7.54 ± 0.47		0.75	9.03 ± 0.01		2.24	9.32 ± 0.06		2.53
	MEM 2	6.83 ± 0.12	2.83 ± 0.77		−4	3.17 ± 1.52		−3.66	9.32 ± 0.02		2.49
	DOR 1	6.85 ± 0.04	4.35 ± 0.27		−2.5	8.36 ± 0.31		1.51	9.38 ± 0.08		2.53
	ETP 0.5 + MEM 2	6.81 ± 0.08	2.88 ± 0.32	0.05	−3.93	2.43 ± 1.07	−0.74	−4.38	8.65 ± 0.97	−0.67	1.84
	ETP 0.5 + DOR 1	6.83 ± 0.10	4.24 ± 1.81	−0.11	−2.59	6.04 ± 1.94	−2.31	−0.79	9.39 ± 0.05	0.07	2.56
	MEM 2 + DOR 1	6.81 ± 0.10	2.67 ± 0.67	−0.16	−4.14	1.36 ± 0.32	−1.81	−5.45	6.40 ± 4.68	−2.93	−0.41
*E. coli* OXA-48 (ARU896)	Growth control	6.35 ± 0.17	8.13 ± 0.18		1.78	8.83 ± 0.11		2.48	9.21 ± 0.02		2.86
	ETP 0.5	6.36 ± 0.17	8.12 ± 0.09		1.76	8.76 ± 0.18		2.4	9.09 ± 0.05		2.73
	ETP 4	6.33 ± 0.14	2.65 ± 0.02		−3.68	5.32 ± 0.03		−1.01	9.18 ± 0.07		2.85
	MEM 0.25	6.35 ± 0.11	7.41 ± 0.23		1.06	8.90 ± 0.02		2.55	9.34 ± 0.07		2.99
	DOR 0.125	6.43 ± 0.12	7.43 ± 0.55		1	8.89 ± 0.05		2.46	9.35 ± 0.12		2.92
	ETP 4 + MEM 0.25	6.41 ± 0.12	2.00 ± 0.06	−0.65	−4.41	4.90 ± 0.07	−0.42	−1.51	9.24 ± 0.02	0.05	2.83
	ETP 0.5 + DOR 0.125	6.42 ± 0.16	4.93 ± 0.60	−2.5	−1.49	6.83 ± 2.49	−1.84	0.41	9.32 ± 0.03	0.23	2.9
	MEM 0.25 + DOR 0.125	6.33 ± 0.09	3.93 ± 0.26	−3.49	−2.4	7.42 ± 0.57	−1.47	1.09	9.35 ± 0.01	0.01	3.02
*K. pneumoniae* KPC-2 (ARU1144)	Growth control	6.68 ± 0.04	8.40 ± 0.06		1.72	9.20 ± 0.02		2.52	9.60 ± 0.14		2.92
	ETP 16	6.80 ± 0.17	2.86 ± 0.07		−3.94	1.80 ± 0.14		−5.00	2.17 ± 0.46		−4.63
	MEM 2	6.77 ± 0.03	4.47 ± 0.78		−2.3	5.43 ± 1.89		−1.34	5.97 ± 1.52		−0.80
	DOR 1	6.66 ± 0.05	4.02 ± 0.08		−2.64	2.96 ± 0.18		−3.70	4.68 ± 0.09		−1.98
	ETP 16 + MEM 2	6.76 ± 0.04	2.63 ± 0.08	−0.23	−4.13	2.09 ± 0.86	0.29	−4.67	4.18 ± 0.14	2.02	−2.58
	ETP 16 + DOR 1	6.65 ± 0.04	2.57 ± 0.07	−0.29	−4.08	1.86 ± 0.36	0.06	−4.79	2.23 ± 1.74	0.07	−4.42
	MEM 2 + DOR 1	6.69 ± 0.01	4.33 ± 0.01	0.3	−2.36	2.72 ± 0.20	−0.24	−3.97	3.48 ± 0.88	−1.2	−3.21
*K. pneumoniae* OXA-48 (ARU735)	Growth control	6.91 ± 0.16	8.37 ± 0.15		1.46	9.07 ±0.04		2.16	9.60 ± 0.14		2.69
	ETP 4	6.85 ± 0.12	5.87 ± 3.61		−0.98	7.51 ± 2.24		0.66	9.60 ± 0.05		2.75
	MEM 2	6.98 ± 0.04	3.89 ± 0.22		−3.09	6.00 ± 0.98		−0.98	9.54 ± 0.19		2.56
	DOR 1	6.83 ± 0.18	6.21 ± 2.60		−0.62	8.34 ± 0.48		1.51	9.54 ± 0.08		2.71
	ETP 4 + MEM 2	6.90 ± 00	3.38 ± 0.55	−0.51	−3.52	4.48 ± 1.93	−1.52	−2.42	7.81 ± 2.32	−1.73	0.1
	ETP 4 + DOR 1	6.80 ± 0.07	4.04 ± 1.61	−1.84	−2.76	6.20 ± 2.93	−1.3	−0.6	9.40 ± 0.17	−0.15	2.6
	MEM 2 + DOR 1	6.88 ± 0.12	3.48 ± 0.22	−0.41	−3.4	4.47 ± 1.83	−1.53	−2.41	7.16 ± 1.83	−2.39	0.28
*K. pneumoniae* OXA-48 (ARU736)	Growth control	6.57 ± 0.15	8.20 ± 0.01		1.63	9.03 ± 0.02		2.46	9.55 ± 0.11		2.98
	ETP 16	6.57 ± 0.06	7.58 ± 0.08		1.01	7.50 ± 0.19		0.93	9.18 ± 0.02		2.61
	MEM 16	6.58 ± 0.03	7.00 ± 0.29		0.42	7.19 ± 0.05		0.61	9.15 ± 0.1		2.57
	DOR 8	6.65 ± 0.09	7.62 ± 0.19		0.97	8.32 ± 0.05		1.67	9.35 ± 0.00		2.7
	DOR 32	6.67 ± 0.28	5.69 ± 0.63		−0.98	4.37 ± 1.86		−2.3	6.66 ± 3.66		−0.1
	ETP 16 + MEM 16	6.63 ± 0.33	6.16 ± 0.08	−0.85	−0.47	6.30 ± 0.35	−0.89	−0.33	9.39 ± 0.04	0.24	2.76
	ETP 16 + DOR 32	6.60 ± 0.06	5.12 ± 0.90	−0.57	−1.48	3.39 ± 1.12	−0.98	−3.21	6.02 ± 4.20	−0.65	−0.58
	MEM 16 + DOR 8	6.67 ± 0.03	6.09 ± 0.18	−0.91	−0.58	5.01 ± 0.76	−2.18	−1.66	8.51 ± 0.26	−0.65	1.84
*K. pneumoniae* NDM-1 (ARU923)	Growth control	6.72 ± 0.08	8.23 ± 0.25		1.51	8.74 ± 0.13		2.02	9.47 ± 0.01		2.75
	ETP 16	6.78 ± 0.02	4.41 ± 0.53		−2.37	7.71 ± 0.25		0.93	9.15 ± 0.02		2.37
	MEM 64	6.85 ± 0.18	2.83 ± 0.33		−4.02	5.92 ± 1.38		−0.93	9.46 ± 0.21		2.61
	DOR 32	6.79 ± 0.18	4.19 ± 0.16		−2.6	7.52 ± 0.35		−0.73	9.43 ± 0.06		2.64
	ETP 16 + MEM 64	6.77 ± 0.08	2.81 ± 0.21	−0.02	−3.96	5.03 ± 0.03	−0.89	−1.74	9.39 ± 0.11	−0.07	2.62
	ETP 16 + DOR 32	6.84 ± 0.10	4.05 ± 0.33	−0.14	−2.79	7.47 ± 0.42	−0.04	0.63	9.48 ± 0.07	0.05	2.64
	MEM 64 + DOR 32	6.97 ± 0.28	2.69 ± 0.41	−0.14	−4.28	4.90 ± 0.25	−1.02	−2.07	9.40 ± 0.12	−0.03	2.43
*K. pneumoniae* NDM-5 (ARU928)	Growth control	5.88 ± 0.35	7.64 ± 0.11		1.76	8.67 ± 0.21		2.79	8.91 ± 0.16		3.03
	ETP 16	5.88 ± 0.59	4.89 ± 0.09		−0.99	5.77 ± 0.78		−0.11	8.82 ± 0.10		2.94
	MEM 64	5.82 ± 0.14	2.72 ± 0.29		−3.1	3.63 ± 1.67		−2.19	5.62 ± 2.89		−0.2
	DOR 32	5. 95 ± 0.41	4.43 ± 0.11		−1.52	4.13 ± 1.41		−1.82	8.83 ± 0.04		2.88
	ETP 16 + MEM 64	5.91 ± 0.43	2.77 ± 0.24	0.05	−3.14	2.73 ± 1.83	−0.9	−3.18	6.71 ± 3.09	1.09	0.8
	ETP 16 + DOR 32	5.93 ± 0.28	3.74 ± 0.18	−0.69	−2.19	3.98 ± 1.30	−0.16	−1.95	8.79 ± 0.13	−0.03	2.86
	MEM 64 + DOR 32	5.82 ± 0.34	2.40 ± 0.17	−0.32	−3.42	2.09 ± 1.01	−1.54	−3.73	5.11 ± 3.27	−0.52	−0.71
*K. pneumoniae* NDM-1+ OXA-48 (ARU724)	Growth control	6. 55 ± 0.01	8.00 ± 0.08		1.45	8.79 ± 0.09		2.24	9.41 ± 0.05		2.86
	ETP 16	6.47 ± 0.01	3.88 ± 0.1		−2.59	6.52 ± 0.04		0.05	9.34 ± 0.01		2.87
	MEM 64	6.47 ± 0.01	3.63 ± 0.13		−2.84	5.09 ± 0.33		−1.38	9.24 ± 0.05		2.77
	DOR 32	6.53 ± 0.11	4.05 ± 0.05		−2.48	6.96 ± 0.15		0.43	9.32 ± 0.01		2.79
	ETP 16 + MEM 64	6.48 ± 0.11	3.51 ± 0.02	−0.12	−2.97	4.91 ± 0.59	−0.18	−1.57	9.22 ± 0.04	−0.02	2.74
	ETP 16 + DOR 32	6.52 ± 0.06	3.86 ± 0.01	−0.02	−2.66	6.28 ± 0.03	−0.24	−0.24	9.33 ± 0.06	0.01	2.81
	MEM 64 + DOR 32	6.48 ± 0.00	3.67 ± 0.01	0.04	−2.81	4.94 ± 0.21	−0.15	−1.54	9.26 ± 0.06	0.02	2.78

^a^ Difference in log_10_ CFU/mL compared to most effective single antibiotic; ^b^ Difference in log_10_ CFU/mL compared to the starting inoculum. Abbreviations: ETP—ertapenem; MEM—meropenem; DOR—doripenem; S—susceptible; I—susceptible with increased exposure; R—resistant.

## Data Availability

Raw data from sequencing are deposited in the NCBI Sequence Read Archive under BioProject accession number PRJNA892093.

## Supplementary Materials

The following are available online at https://www.mdpi.com/article/10.3390/antibiotics11111646/s1, Figure S1. Time-lapse microscopy and spot assay results at 24 h for double-carbapenem combinations (ertapenem, meropenem, and doripenem) against clinical isolates. The isolates are color-coded based on carbapenemase type. Dark grey represents growth (>ca 10^6^ CFU/mL) in time-lapse microscopy at 6 and 24 h, as determined with the algorithms and predefined cut-offs for BCA (>8) and SESAmax (>5.8). Light grey depicts growth only at 24 h, and white boxes represent no growth at 6 or 24 h. Bacterial growth at 24 h, as determined with the spot assay is presented in log10 CFU/mL. No visible growth was set to 1 log10 CFU/mL (LOD = 2 log10 CFU/mL). Combinations showing enhanced effects compared to the most active single antibiotic in the time-lapse assay are marked with a thick orange outline. Combinations showing reduced effects are marked with a thick black outline. Synergistic (*) and antagonistic effects (+) with the combination are also indicated. When the synergistic effect was also bactericidal, the log10 CFU/mL value is marked in bold. Table S1. Fisher’s Exact Test (GraphPad Prism version 9.4.0) testing for associations between synergistic effect in the spot assay and antibiotic susceptibility to meropenem or doripenem, and the presence of specific carbapenemase genes. Statistically significant (*p* < 0.05) results are marked in yellow. Table S2. Mutants isolated from antibiotic-containing plates following 24-hour time-kill experiments with *E. coli* ATCC 25922 wild-type and constructed carbapenemase-producing strains. The time-kill regimen in which the mutant arose and the antibiotic-containing plate on which it was selected is presented. MIC values for ertapenem, meropenem, and doripenem are presented for mutants. The fold increase in MIC compared to the parental strain is presented in parentheses. Bacterial concentration (CFU/mL) on antibiotic plates and non-selective plates (viable count) and mutant frequency are presented. Growth rates of mutants are relative to the respective parental strain. Table S3. Whole genome sequencing results of mutants with decreased susceptibility to carbapenems isolated following time-kill experiments with *E. coli* ATCC 25922 wild-type and constructed carbapenemase-producing strains. Point mutations, amino acid changes, and structural variations in *E. coli* ATCC 25922 mutants are presented. Table S4. Primers used in the study. Gradient PCR (Thermo Scientific™ Phusion™ High-Fidelity DNA Polymerase) was used for amplification.
