# Supplementary material for: Evaluation of In Vitro Activity of Double-Carbapenem Combinations against KPC-2-, OXA-48- and NDM-Producing Escherichia coli and Klebsiella pneumoniae
Source: Antibiotics (Basel). 2022 Nov 17;11(11):1646. doi: 10.3390/antibiotics11111646 (PMC9686504; doi:10.3390/antibiotics11111646)
Supplement: Supplementary file 1 [file antibiotics-11-01646-s001.zip › antibiotics-1955157-supplementary.pdf]

Supplementary data

Evaluation of *in vitro* activity of double-carbapenem combinations against KPC-2-, OXA-48- and NDM-producing *Escherichia coli* and *Klebsiella pneumoniae*

Lisa Allander[1], Karin Vickberg[1], Pernilla Lagerbäck[1], Linus Sandegren[2], Thomas Tängdén[1]

[1] Department of Medical Sciences, Uppsala University, Sweden.

[2] Department of Medical Biochemistry and Microbiology, Uppsala University, Sweden.

Table S1. Fisher's Exact Test (GraphPad Prism version 9.4.0) testing for associations between synergistic effect in the spot assay and antibiotic susceptibility to meropenem or doripenem, and the presence of specific carbapenemase genes. Statistically significant (P < 0.05) results are marked in yellow.

Carbapenem susceptibility							
Species	Combination	Meropenem susceptibility	Synergy	No synergy	P-value	Odds ratio	95% CI
E. coli	ETP + MEM	S, I	10	4	0.0006	+infinity	5.010 – +infinity
		R	0	10			
	ETP + DOR	S, I	10	4	0.0006	+infinity	5.010 – +infinity
		R	0	10			
	MEM + DOR	S, I	9	5	0.002	+infinity	3.807 – +infinity
		R	0	10			
K. pneumoniae	ETP + MEM	S, I	6	1	0.0002	114.0	6.944 – 1318
		R	1	19			
	ETP + DOR	S, I	2	5	0.2692	3.600	0.4477 – 26.01
		R	2	18			
	MEM + DOR	S, I	4	3	0.1751	4.000	0.7459 – 19.30
		R	5	15			
Species	Combination	Doripenem susceptibility	Synergy	No synergy	P-value	Odds ratio	95% CI
E. coli	ETP + MEM	S, I	10	3	0.0002	+infinity	6.671 – +infinity
		R	0	11			
	ETP + DOR	S, I	9	4	0.0045	22.50	2.112 – 265.5
		R	1	10			
	MEM + DOR	S, I	8	5	0.0131	16.00	1.563 – 191.5
		R	1	10			
K. pneumoniae	ETP + MEM	S, I	5	0	0.0003	+infinity	6.339 – +infinity
		R	2	20			
	ETP + DOR	S, I	2	3	0.1444	6.667	0.7369 – 50.35
		R	2	20			
	MEM + DOR	S, I	3	2	0.2950	4.000	0.6433 – 25.35
		R	6	16			

Carbapenemase type							
Species	Combination	KPC-2 vs. Non-KPC-2	Synergy	No synergy	P-value	Odds ratio	95% CI
E. coli	ETP + MEM				NA		
	ETP + DOR				NA		
	MEM + DOR				NA		
K. pneumoniae	ETP + MEM	KPC-2	1	9	0.2040	0.2037	0.01619 – 2.032
		Non-KPC-2	6	11			
	ETP + DOR	KPC-2	1	9	> 0.9999	0.5185	0.03635 – 4.055
		Non-KPC-2	3	14			
	MEM + DOR	KPC-2	1	9	0.0912	0.1250	0.01029 – 1.112
		Non-KPC-2	8	9			
Species	Combination	OXA-48 vs. Non-OXA-48	Synergy	No synergy	P-value	Odds ratio	95% CI

E. coli	ETP + MEM	OXA-48	8	2	0.0027	24.00	2.515 – 148.1
		Non-OXA-48	2	12			
	ETP + DOR	OXA-48	8	2	0.0027	24.00	2.515 – 148.1
		Non-OXA-48	2	12			
	MEM + DOR	OXA-48	7	3	0.0104	14.00	1.699 – 81.13
		Non-OXA-48	2	12			
K. pneumoniae	ETP + MEM	OXA-48	4	3	0.0496	7.556	1.181 – 39.82
		Non-OXA-48	3	17			
	ETP + DOR	OXA-48	3	4	0.0419	14.25	1.536 – 191.4
		Non-OXA-48	1	19			
	MEM + DOR	OXA-48	5	2	0.0235	10.00	1.541 – 57.98
		Non-OXA-48	4	16			
Species	Combination	NDM vs. Non-NDM	Synergy	No synergy	P-value	Odds ratio	95% CI
E. coli	ETP + MEM	NDM	0	10	0.0006	0.000	0.000 – 0.1996
		Non-NDM	10	4			
	ETP + DOR	NDM	0	10	0.0006	0.000	0.000 – 0.1996
		Non-NDM	10	4			
	MEM + DOR	NDM	0	10	0.002	0.000	0.000 – 0.2627
		Non-NDM	9	5			
K. pneumoniae	ETP + MEM	NDM	2	8	0.6784	0.6000	0.1019 – 3.296
		Non-NDM	5	12			
	ETP + DOR	NDM	0	10	0.2638	0.000	0.000 – 1.835
		Non-NDM	4	13			
	MEM + DOR	NDM	3	7	>0.9999	0.7857	0.1725 – 4.546
		Non-NDM	6	11			

Abbreviations: ETP, ertapenem; MEM, meropenem; DOR, doripenem; S, susceptible; I, susceptible with increased exposure; R, resistant; CI, confidence interval; NA, not applicable because of low number of KPC-2 producing *E. coli* (n=4).

Table S2. Mutants isolated from antibiotic-containing plates following 24-hour time-kill experiments with *E. coli* ATCC 25922 wild-type and constructed carbapenemase-producing strains. The time-kill regimen in which the mutant arose and the antibiotic-containing plate on which it was selected are presented. MIC values for ertapenem, meropenem and doripenem are presented for mutants. The fold-increase in MIC compared to the parental strain is presented in parentheses. Bacterial concentration (CFU/mL) on antibiotic plates and non-selective plates (viable count), and mutant frequency are presented. Growth rates of mutants are relative to the respective parental strain.

***E. coli* ATCC 25922 mutants**	**Time-kill regimen**	**Concentration in plate**	**MIC (mg/L)**			**Bacterial concentration (CFU/mL)**		**Mutant frequency**	**Relative growth rate (± SD)**
			**ETP**	**MEM**	**DOR**	**Antibiotic plate**	**Viable count**		
Wild-type clone 1	0.5x MIC ETP	4x MIC ETP	0.5 (x64)	0.125 (x8)	0.062 (x2)	1×10^2	5.30×10^8	1.89×10^{-7}	0.56 ± 0.01
bla$_{OXA-48}$ clone 1	Growth control	4x MIC ETP	2 (x16)	1 (x32)	2 (x32)	1×10^2	1.74×10^9	5.75×10^{-8}	0.91 ± 0.05
bla$_{OXA-48}$ clone 2	1x MIC ETP+MEM	4x MIC ETP	4 (x32)	1 (x32)	1 (x16)	5×10^1	1.72×10^9	2.91×10^{-8}	0.79 ± 0.06
bla$_{OXA-48}$ clone 3	0.5x MIC ETP+MEM	4x MIC MEM	2 (x16)	1 (x32)	2 (x32)	2×10^2	2.40×10^9	8.33×10^{-8}	1.01 ± 0.03
bla$_{NDM-1}$ clone 1	1x MIC DOR	4x MIC DOR	64 (x4)	32 (x2)	32 (x2)	NA	1.56×10^9	NA	0.94 ± 0.04

Abbreviations: ETP, ertapenem; MEM, meropenem; DOR, doripenem. NA, not available because no single colonies were detected but low growth with colonies fused together occurred at 10-fold dilution.

Table S3. Whole genome sequencing results of mutants with decreased susceptibility to carbapenems isolated following time-kill experiments with *E. coli* ATCC 25922 wild-type and constructed carbapenemase-producing strains. Point mutations, amino acid changes and structural variations in *E. coli* ATCC 25922 mutants are presented.

Parental strain	*E. coli* ATCC 25922 mutants	Genetic changes	Function
ARU961	Wild-type clone 1	–	–
ARU1028	*bla*$_{OXA-48}$ clone 1	IS3 inserted 169 nucleotides upstream *bla*$_{OXA-48}$ start codon	–
ARU1028	*bla*$_{OXA-48}$ clone 2	*phoR* 217G>T, Glu73*	phoR: phosphate regulon sensor histidine kinase PhoR
		rclR 719G>A, Arg240Gln	rclR: AraC family transcriptional regulator
ARU1028	*bla*$_{OXA-48}$ clone 3	*Gene DR76_RS09145* 1672G>T, Glu558*	Gene DR76_RS09145: sigma-54-dependent transcriptional regulator
		yeaQ 210G>T	yeaQ: GlsB/YeaQ/YmgE family stress response membrane protein
		trpS 918G>T, Met306Ile<br>*trpS* 956G>T, Arg319Leu	trpS: tryptophan-tRNA ligase
		T>C,176 nucleotides upstream of *bla*$_{OXA-48}$ start codon	
ARU1027	*bla*$_{NDM-1}$ clone 1	–	–

Abbreviations: *, premature stop codon.

Table S4. Primers used in the study. Gradient PCR (Thermo Scientific™ Phusion™ High-Fidelity DNA Polymerase) was used for amplifications.

**Application**	**Sequence (5´→3´)**
Amplification of cat-sacB cassette with regions homologous to *bgl* in ATCC 25922	**Forward:** GCTCGATAAACTGCTGGCAGAAAAAGATAGC GATAAATAATTCACCAGACAAATCCCAAT<br><br>**Reverse:** GACTGTTCTGAATGCGACGATATTTAAGGTGC TTTATTGGAATATCCCTTTATGGTGCAAAG
Amplification of *bla*$_{NDM-1}$ with homologies to *cat-sacB*	**Forward:** AGCCGGATTAATAATCTGGCTTTTTATATTCTC TGTCGACTTGAATTCGCCCCATATTTT<br><br>**Reverse:** AAAGCCCCGAGCGGTTAAACTCAGGGCTTTAT TTGAGCTCTCAGCGCAGCTTGTCGGCCA
Amplification of *bla*$_{KPC-2}$ with homologies to *cat-sacB*	**Forward:** AGCCGGATTAATAATCTGGCTTTTTATATTCTCTG TCGACTAGGTGAAGTTCTGGGCAGT<br><br>**Reverse:** AAAGCCCCGAGCGGTTAAACTCAGGGCTTTATTT GAGCTCCTAGGGAATAATTTTTTCCT
Amplification of *bla*$_{OXA-48}$ with homologies to *cat-sacB*	**Forward:** AGCCGGATTAATAATCTGGCTTTTTATATTCTCTGT CGACGGTCAGTTTTCAGTTGGTGT<br><br>**Reverse:** AAAGCCCCGAGCGGTTAAACTCAGGGCTTTATTTGA GCTCGCGGTGGTGGGCCAATAGAT
Screening of insertion site ATCC 25922 *bgl*	**Forward:** CGCTGCCAGAATATTTGTGA<br><br>**Reverse:** CGCCTTTTCTAATAGCTCAA

(a) Ertapenem + meropenem

KPC-2 | OXA-48 | NDM-1 | NDM-5 | NDM-1 + OXA-48

Clinical *E. coli* isolates

Meropenem concentration (mg/L)

Ertapenem concentration (mg/L)

ARU887	0	2	16	64
0	8	9	1	2
0.5	8	8	1	1
4	8	8	1	1
16	8	8	1	1

ARU888	0	0.25	2	16
0	9	8	8	9
0.5	8	9	9	3*
4	8	8	9	1*
16	9	9	8	1*

ARU894	0	0.25	2	16
0	9	8	8	8
0.5	8	8	8	2*
4	8	8	8	6*
16	8	8	8	4*

ARU1141	0	2	16	64
0	7	7	8	8
0.5	7	8	8	7
4	7	7	8	7
16	9	8	7	8

ARU716	0	0.25	2	16
0	9	9	9	1
0.5	9	8	8	1
4	8	8	8	1
16	8	8	5*	1

ARU722	0	0.25	2	16
0	9	8	9	1
0.5	8	8	7	1
4	8	8	7	1
16	8	8	8	1

ARU889	0	2	16	64
0	9	9	1	1
0.5	9	9	1	1
4	8	9	1	1
16	1	1	1	1

ARU890	0	2	16	64
0	8	9	1	1
0.5	8	8	1	1
4	7	8	1	1
16	5	1*	1	1

ARU891	0	0.25	2	16
0	9	9	9	1
0.5	8	8	1*	1
4	1	1	1	1
16	1	1	1	1

ARU896	0	0.25	2	16
0	8	8	1	2
0.5	8	7	1	1
4	8	1*	1	1
16	1	1	1	1

ARU898	0	0.25	2	16
0	9	9	8	1
0.5	8	8	9	1
4	7	7	1*	1
16	7	1*	1*	1

ARU903	0	2	16	64
0	9	9	1	1
0.5	8	8	1	1
4	9	1*	1	1
16	1	1	1	1

ARU991	0	0.25	2	16
0	8	9	9	1
0.5	9	8	8	1
4	8	8	1*	1
16	1	1	1	1

ARU992	0	0.25	2	16
0	9	8	9	1
0.5	9	9	7*	1
4	8	8	8	1
16	8	7	1*	1

ARU711	0	2	16	64
0	8	8	8	9
0.5	9	8	9	8
4	9	9	8	9
16	9	8	9	8

ARU713	0	2	16	64
0	8	8	9	9
0.5	9	8	8	9
4	9	8	8	8
16	9	8	9	9

ARU714	0	2	16	64
0	8	9	9	9
0.5	8	9	8	8
4	8	9	9	8
16	8	8	9	8

ARU892	0	2	16	64
0	8	9	8	9
0.5	9	9	8	9
4	8	8	8	9
16	9	8	8	8

ARU709	0	2	16	64
0	8	8	8	8
0.5	8	8	8	8
4	8	9	8	8
16	8	8	9	9

ARU717	0	2	16	64
0	8	8	8	8
0.5	9	8	9	9
4	8	8	8	8
16	8	9	8	9

ARU910	0	2	16	64
0	9	8	8	8
0.5	9	8	9	9
4	8	9	8	8
16	8	9	8	8

ARU912	0	2	16	64
0	9	8	8	8
0.5	8	8	8	8
4	9	8	8	8
16	8	8	8	8

ARU913	0	2	16	64
0	9	8	8	8
0.5	8	7	8	8
4	8	8	8	8
16	8	8	8	8

ARU917	0	2	16	64
0	9	8	7	7
0.5	7	7	8	8
4	7	7	8	8
16	8	7	8	8

Clinical *K. pneumoniae* isolates

Meropenem concentration (mg/L)

Ertapenem concentration (mg/L)

ARU737	0	2	16	64
0	8	8	9	8
0.5	9	9	8	8
4	9	9	8	8
16	9	8	8	8

ARU869	0	2	16	64
0	9	9	8	8
0.5	8	8	8	9
4	8	9	9	8
16	9	8	8	9

ARU871	0	2	16	64
0	8	8	8	7
0.5	7	8	8	8
4	7	8	8	8
16	9	8	8	8

ARU919	0	2	16	64
0	8	8	8	8
0.5	8	9	9	8
4	9	9	8	7
16	9	8	8	8

ARU920	0	2	16	64
0	9	9	8	8
0.5	8	9	8	8
4	9	8	9	8
16	8	8	7	8

ARU1011	0	2	16	64
0	8	8	7	1
0.5	8	9	8	1
4	8	9	8	1
16	8	8	8	1

ARU1015	0	2	16	64
0	8	8	8	8
0.5	8	8	8	8
4	7	8	7	8
16	8	7	7	7

ARU1016	0	2	16	64
0	8	8	8	7
0.5	8	8	8	7
4	8	8	8	8
16	7	7	8	8

ARU1019	0	2	16	64
0	8	8	8	7
0.5	8	8	7	8
4	8	9	8	7
16	8	8	8	8

ARU1144	0	0.25	2	16
0	9	8	8	1
0.5	9	8	8	1
4	8	8	8	1
16	8	1*	1*	1

ARU731	0	2	16	64
0	8	8	8	1
0.5	8	8	7	1
4	8	8	7	1
16	7	8	7	1

ARU734	0	2	16	64
0	9	8	1	1
0.5	9	8	1	1
4	8	8	1	1
16	8	7	1	1

ARU735	0	0.25	2	16
0	8	9	8	1
0.5	9	8	8	1
4	8	8	1*	1
16	1	1	1	1

ARU736	0	2	16	64
0	8	8	7	1
0.5	8	9	7	1
4	8	8	8	1
16	7	8	7	1

ARU873	0	0.25	2	16
0	8	8	7	7
0.5	8	8	7	1*
4	8	7	8	1*
16	8	8	7	1*

ARU874	0	0.25	2	16
0	9	9	9	1
0.5	9	7*	8	8†
4	8	8	1*	1
16	1	1	1	8†

ARU1005	0	0.25	2	16
0	8	8	8	1
0.5	8	8	8	1
4	8	7	5*	1
16	8	8	1*	1

ARU725	0	2	16	64
0	8	8	9	8
0.5	9	8	9	9
4	8	9	8	8
16	8	9	8	8

ARU726	0	2	16	64
0	8	8	8	8
0.5	9	9	8	8
4	8	8	7	8
16	7	8	8	8

ARU884	0	2	16	64
0	9	8	9	1
0.5	8	8	8	1
4	8	8	8	1
16	8	8	9	1

ARU923	0	2	16	64
0	8	8	8	8
0.5	8	7	8	8
4	8	8	9	8
16	7	9†	8	1*

ARU601	0	2	16	64
0	8	8	8	8
0.5	8	8	8	7
4	8	8	8	7
16	8	7	8	7

ARU733	0	2	16	64
0	8	8	9	8
0.5	8	8	7	8
4	8	8	8	9
16	7	7	8	7

ARU928	0	2	16	64
0	8	7	6	7
0.5	7	6	6	5*
4	7	6	5	1*
16	6	6	5	1*

ARU724	0	2	16	64
0	7	8	8	8
0.5	8	9	8	8
4	8	8	8	8
16	7	7	8	8

ARU879	0	2	16	64
0	8	9	8	8
0.5	8	9	8	8
4	8	8	8	9
16	8	8	8	9

ARU882	0	2	16	64
0	8	8	7	9
0.5	8	8	8	8
4	8	8	8	8
16	7	7	8	8

(b) Ertapenem + doripenem

KPC-2 | OXA-48 | NDM-1 | NDM-5 | NDM-1 + OXA-48

Clinical *E. coli* isolates

Doripenem concentration (mg/ L)

Ertapenem concentration (mg/ L)

ARU887	0	0.125	1	8
0	8	8	8	1
0.5	8	8	8	1
4	8	9	9	1
16	8	8	8	1

ARU888	0	0.125	1	8
0	9	9	9	8
0.5	9	8	9	9
4	9	9	8	1*
16	9	8	8	3*

ARU894	0	0.125	1	8
0	9	8	8	8
0.5	9	8	8	9
4	8	8	9	7
16	8	9	8	7

ARU1141	0	1	8	32
0	7	8	8	1
0.5	8	7	7	1
4	7	7	7	1
16	7	8	1*	1

ARU716	0	0.125	1	8
0	8	8	8	8
0.5	8	8	8	9
4	9	8	8	1*
16	9	8	8	1*

ARU722	0	1	8	32
0	8	8	1	1
0.5	8	8	1	1
4	8	1*	1	1
16	9	1*	1	1

ARU889	0	0.125	1	8
0	9	9	9	9
0.5	9	9	9	8
4	9	8	9	1*
16	8	8	9	1*

ARU890	0	0.125	1	8
0	9	9	8	8
0.5	9	8	9	7
4	8	8	8	8
16	8	8	9	1*

ARU891	0	0.125	1	8
0	9	9	9	1
0.5	9	8	6*	1
4	1	1	1	1
16	1	1	1	1

ARU896	0	0.125	1	8
0	8	8	1	1
0.5	8	7	1	1
4	1	1	1	1
16	1	1	1	1

ARU898	0	0.125	1	8
0	8	8	8	1
0.5	9	8	8	1
4	8	7	8	1
16	1	1	1	1

ARU903	0	0.125	1	8
0	9	8	9	3
0.5	8	8	9	1*
4	9	8	8	1*
16	4	1*	1*	1*

ARU991	0	0.125	1	8
0	8	9	9	1
0.5	8	8	9	1
4	8	9	1*	1
16	1	1	1	1

ARU992	0	1	8	32
0	8	9	1	1
0.5	8	8	1	1
4	9	5*	1	1
16	8	1*	1	1

ARU711	0	1	8	32
0	9	8	8	9
0.5	9	8	9	8
4	8	8	8	9
16	9	8	7	8

ARU713	0	1	8	32
0	8	8	9	9
0.5	9	9	9	8
4	8	9	8	8
16	9	9	8	8

ARU714	0	1	8	32
0	9	9	8	9
0.5	9	8	9	8
4	9	9	8	9
16	9	9	8	9

ARU892	0	1	8	32
0	8	9	9	8
0.5	9	9	9	8
4	9	8	8	9
16	9	9	8	9

ARU709	0	1	8	32
0	8	8	8	9
0.5	9	9	8	8
4	9	8	8	8
16	8	8	9	8

ARU717	0	1	8	32
0	9	8	8	8
0.5	9	8	9	8
4	8	9	8	9
16	8	8	8	8

ARU910	0	1	8	32
0	9	8	8	8
0.5	8	8	8	9
4	9	8	8	8
16	8	8	8	8

ARU912	0	1	8	32
0	9	8	8	8
0.5	8	8	7	9
4	8	8	8	9
16	9	8	9	8

ARU913	0	1	8	32
0	9	9	8	8
0.5	8	8	8	7
4	8	8	8	8
16	8	8	8	8

ARU917	0	1	8	32
0	8	7	8	8
0.5	7	7	7	7
4	7	7	6	6
16	7	7	7	8

Clinical *K. pneumoniae* isolates

Doripenem concentration (mg/ L)

Ertapenem concentration (mg/ L)

ARU737	0	1	8	32
0	8	9	9	7
0.5	8	8	8	7
4	9	8	8	8
16	8	9	8	7

ARU869	0	1	8	32
0	8	9	9	9
0.5	9	9	8	8
4	8	9	8	8
16	8	8	8	9

ARU871	0	1	8	32
0	8	8	8	9
0.5	8	9	8	8
4	8	9	8	8
16	9	8	9	8

ARU919	0	1	8	32
0	8	8	7	8
0.5	7	8	7	7
4	8	8	7	8
16	8	9	8	8

ARU920	0	1	8	32
0	7	8	9	8
0.5	9	8	8	8
4	8	8	8	9
16	8	8	8	8

ARU1011	0	1	8	32
0	8	8	8	1
0.5	8	7	7	1
4	7	7	8	1
16	8	8	8	1

ARU1015	0	1	8	32
0	8	9	8	8
0.5	8	8	8	8
4	8	8	8	8
16	7	8	7	8

ARU1016	0	1	8	32
0	8	8	8	8
0.5	8	8	9	9
4	8	8	8	8
16	8	8	8	8

ARU1019	0	1	8	32
0	8	8	8	8
0.5	8	8	8	8
4	9	8	9	8
16	9	8	8	8

ARU1144	0	0.125	1	8
0	9	8	8	1
0.5	8	8	8	1
4	8	8	8	1
16	8	1*	1*	1

ARU731	0	1	8	32
0	8	8	8	1
0.5	9	8	8	1
4	8	8	8	1
16	8	8	9	1

ARU734	0	1	8	32
0	9	9	8	1
0.5	8	8	8	1
4	9	9	1*	1
16	8	7	1*	1

ARU735	0	0.125	1	8
0	9	8	8	1
0.5	9	8	8	1
4	8	8	9	1
16	1	1	1	1

ARU736	0	1	8	32
0	9	8	8	3
0.5	8	8	8	1*
4	9	8	8	1*
16	8	8	7	1*

ARU873	0	1	8	32
0	8	8	8	1
0.5	9	9	1*	1
4	7	8	1*	1
16	1	1	1	1

ARU874	0	1	8	32
0	8	8	1	1
0.5	8	8	1	1
4	8	8	1	1
16	1	1	1	1

ARU1005	0	1	8	32
0	9	8	8	1
0.5	8	8	8	1
4	8	8	8	1
16	7	8	7	1

ARU725	0	1	8	32
0	9	9	8	8
0.5	9	8	9	9
4	9	8	8	8
16	9	9	9	9

ARU726	0	1	8	32
0	8	9	8	8
0.5	8	8	8	8
4	8	9	9	8
16	8	8	8	8

ARU884	0	1	8	32
0	9	8	8	9
0.5	9	8	8	9
4	8	9	8	8
16	9	8	8	8

ARU923	0	1	8	32
0	8	7	8	8
0.5	8	8	8	8
4	8	8	8	8
16	7	8	8	8

ARU601	0	1	8	32
0	8	8	8	8
0.5	8	8	7	7
4	8	8	8	8
16	8	8	7	8

ARU733	0	1	8	32
0	8	8	8	8
0.5	8	8	8	8
4	8	8	7	8
16	8	8	7	8

ARU928	0	1	8	32
0	8	7	7	7
0.5	7	6	6	8
4	7	7	7	7
16	8	7	8	8

ARU724	0	1	8	32
0	8	9	8	8
0.5	9	8	9	8
4	8	8	8	8
16	7	8	7	8

ARU879	0	1	8	32
0	9	9	8	9
0.5	8	9	9	8
4	8	9	9	8
16	9	9	8	8

ARU882	0	1	8	32
0	9	8	9	8
0.5	8	9	8	8
4	8	8	8	9
16	8	8	9	8

(c) Meropenem + doripenem

KPC-2 | OXA-48 | NDM-1 | NDM-5 | NDM-1 + OXA-48

Clinical *E. coli* isolates

Meropenem concentration (mg/L)

Doripenem concentration (mg/L)

ARU887	0	0.25	2	16
0	7	8	9	1
0.125	8	7	8	1
1	7	8	8	1
8	1	1	1	1

ARU888	0	0.25	2	16
0	9	8	8	9
0.125	8	8	9	9
1	8	9	9	9
8	8	8	8	6*

ARU894	0	0.25	2	16
0	9	9	8	8
0.125	8	8	8	8
1	8	8	9	8
8	8	8	8	9

ARU1141	0	2	16	64
0	9	8	8	1
1	7	8	8	1
8	8	8	**1***	1
32	1	1	1	1

ARU716	0	0.25	2	16
0	8	8	8	1
0.125	8	9	8	1
1	9	8	8	1
8	9	5*	**1***	1

ARU722	0	0.25	2	16
0	8	8	8	1
1	8	7	**1***	1
8	1	1	1	1
32	1	1	1	1

ARU889	0	0.25	2	16
0	9	9	9	1
0.125	9	9	9	1
1	8	8	8	1
8	8	9	8	1

ARU890	0	0.25	2	16
0	8	8	9	1
0.125	8	8	8	1
1	9	8	8	1
8	9	8	**1***	1

ARU891	0	0.25	2	16
0	9	9	8	1
0.125	9	9	**1***	1
1	8	8	**1***	1
8	1	1	1	1

ARU896	0	0.25	2	16
0	8	8	1	1
0.125	8	7	1	1
1	1	1	1	1
8	1	1	1	1

ARU898	0	0.25	2	16
0	8	9	8	1
0.125	8	8	8	1
1	7	8	**1***	1
8	1	1	1	1

ARU903	0	0.25	2	16
0	8	8	8	1
0.125	8	8	8	1
1	9	9	8	1
8	8	5*	**1***	1

ARU991	0	0.25	2	16
0	8	8	8	1
0.125	8	8	8	1
1	8	8	8	1
8	1	1	1	1

ARU992	0	0.25	2	16
0	9	9	9	1
1	8	8	**1***	1
8	1	1	1	1
32	1	1	1	1

ARU711	0	2	16	64
0	8	8	9	9
1	8	8	9	8
8	7	8	8	8
32	9	8	8	8

ARU713	0	2	16	64
0	9	8	9	8
1	9	8	8	9
8	8	7	8	8
32	9	9	8	9

ARU714	0	2	16	64
0	8	9	9	9
1	9	9	8	8
8	8	9	8	8
32	8	9	8	8

ARU892	0	2	16	64
0	9	9	9	8
1	9	8	8	9
8	9	8	8	8
32	8	9	9	8

ARU709	0	2	16	64
0	8	9	8	8
1	8	7	9	8
8	8	8	8	8
32	8	9	9	8

ARU717	0	2	16	64
0	9	8	8	9
1	8	8	8	8
8	8	8	8	8
32	8	8	9	9

ARU913	0	2	16	64
0	8	8	7	9
1	8	7	7	8
8	8	8	8	8
32	8	8	8	9

ARU910	0	2	16	64
0	8	8	8	8
1	8	9	8	8
8	7	8	8	8
32	8	8	8	9

ARU912	0	2	16	64
0	9	8	9	8
1	9	8	8	8
8	9	8	8	8
32	8	8	8	8

ARU917	0	2	16	64
0	8	8	7	7
1	7	7	8	7
8	8	7	8	8
32	7	7	7	8

Clinical *K. pneumoniae* isolates

Meropenem concentration (mg/L)

Doripenem concentration (mg/L)

ARU737	0	2	16	64
0	9	8	8	8
1	9	8	9	8
8	9	8	8	8
32	8	7	8	8

ARU869	0	2	16	64
0	8	8	9	8
1	9	8	8	8
8	8	8	8	8
32	8	8	9	8

ARU871	0	2	16	64
0	8	9	8	9
1	9	8	8	8
8	8	9	7	8
32	8	8	8	7

ARU919	0	2	16	64
0	9	9	8	8
1	8	7	7	8
8	8	7	8	8
32	8	7	8	8

ARU920	0	2	16	64
0	8	8	9	8
1	8	9	9	8
8	8	8	7	7
32	8	8	8	8

ARU1011	0	2	16	64
0	8	8	8	7
1	8	8	8	5*
8	8	7	7	**2***
32	1	1	1	1

ARU1015	0	2	16	64
0	8	8	7	8
1	8	8	8	8
8	8	8	7	8
32	7	7	7	8

ARU1016	0	2	16	64
0	8	8	8	8
1	8	8	9	8
8	8	8	7	7
32	7	8	8	8

ARU1019	0	2	16	64
0	8	8	8	7
1	8	8	8	7
8	8	8	7	7
32	8	8	8	8

ARU1144	0	0.25	2	16
0	8	7	7	1
0.125	7	7	8	1
1	8	8	8	1
8	1	1	1	1

ARU731	0	2	16	64
0	8	9	8	1
1	9	9	8	1
8	8	8	4*	1
32	1	1	1	1

ARU734	0	2	16	64
0	9	8	1	1
1	9	9	1	1
8	1	1	1	1
32	1	1	1	1

ARU735	0	0.25	2	16
0	9	8	8	1
0.125	8	8	8	1
1	8	8	**1***	1
8	1	1	1	1

ARU736	0	2	16	64
0	8	9	8	1
1	9	8	9	1
8	8	8	**1***	1
32	1	1	1	1

ARU873	0	0.25	2	16
0	8	8	8	1
1	8	8	8	1
8	7	6	**1***	1
32	1	1	1	1

ARU874	0	0.25	2	16
0	8	8	8	9
1	7	8	8	**1***
8	8	8	**1***	**1***
32	1	1	1	1

ARU1005	0	0.25	2	16
0	8	8	7	1
1	8	8	8	1
8	8	8	8	1
32	1	1	1	1

ARU725	0	2	16	64
0	9	9	8	8
1	9	8	8	8
8	8	9	9	8
32	8	8	9	9

ARU726	0	2	16	64
0	8	8	9	8
1	9	8	8	8
8	8	8	8	8
32	8	7	8	8

ARU884	0	2	16	64
0	9	8	8	1
1	8	8	8	1
8	8	8	8	1
32	9	8	8	1

ARU923	0	2	16	64
0	8	7	8	8
1	8	8	8	8
8	8	7	7	8
32	8	8	7	**1***

ARU601	0	2	16	64
0	8	7	8	8
1	7	8	7	8
8	9	8	9	8
32	7	7	7	7

ARU733	0	2	16	64
0	8	8	8	7
1	8	8	8	8
8	8	8	8	8
32	8	8	7	8

ARU928	0	2	16	64
0	7	6	7	6
1	7	7	6	8^{+}
8	7	8^{+}	6	7
32	7	8^{+}	6	**1***

ARU724	0	2	16	64
0	8	8	7	7
1	8	8	7	**1***
8	8	8	7	**1***
32	8	7	8	**1***

ARU879	0	2	16	64
0	8	9	8	8
1	8	9	8	8
8	9	9	8	9
32	9	9	8	8

ARU882	0	2	16	64
0	8	8	8	8
1	8	8	7	8
8	8	8	8	7
32	7	8	7	7

Figure S1. Time-lapse microscopy and spot assay results at 24 hours for double-carbapenem combinations (ertapenem, meropenem and doripenem) against clinical isolates. The isolates are color-coded based on carbapenemase type. Dark grey represents growth (> ca 10^6 CFU/mL) in time-lapse microscopy at 6 and 24 hours, as determined with the algorithms and predefined cut-offs for BCA (> 8) and $SESA_{max}$ (> 5.8). Light grey depicts growth only at 24 hours and white boxes represent no growth at 6 or 24 h. Bacterial growth at 24 hours, as determined with the spot assay is presented in log_{10} CFU/mL. No visible growth was set to 1 log_{10} CFU/mL (LOD = 2 log_{10} CFU/mL). Combinations showing enhanced effect compared to the most active single antibiotic in the time-lapse assay are marked with a thick orange outline. Combinations showing reduced effects are marked with a thick black outline. Synergistic (*) and antagonistic effects ($^{+}$) with the combination are also indicated. When the synergistic effect was also bactericidal, the log_{10} CFU/mL value is marked in bold.
